# Evaluation of capsular contracture following immediate prepectoral versus subpectoral direct-to-implant breast reconstruction

**DOI:** 10.1038/s41598-020-58094-4

**Published:** 2020-01-24

**Authors:** Nikhil Sobti, Rachel E. Weitzman, Kassandra P. Nealon, Rachel B. Jimenez, Lisa Gfrerer, David Mattos, Richard J. Ehrlichman, Michele Gadd, Michelle Specht, William G. Austen, Eric C. Liao

**Affiliations:** 1 Division of Plastic and Reconstructive Surgery, Massachusetts General Hospital, Boston, MA USA; 2 Division of Radiation Oncology, Massachusetts General Hospital, Boston, MA USA; 3 Division of Surgical Oncology, Massachusetts General Hospital, Boston, MA USA

**Keywords:** Outcomes research, Surgical oncology

## Abstract

Capsular contracture is a common adverse outcome following implant breast reconstruction, often associated with radiation treatment. The authors hypothesize that muscle fibrosis is the main contributor of breast reconstruction contracture after radiation. Retrospective chart review identified patients that underwent DTI reconstruction with pre-or post-operative breast irradiation. Signs of capsular contracture were assessed using clinic notes and independent graders reviewing two-dimensional images and anatomic landmarks. Capsular contracture rate was greater in the subpectoral vs. prepectoral group (n = 28, 51.8% vs. n = 12, 30.0%, p = 0.02). When compared to prepectoral DTI reconstruction in irradiated patients, subpectoral implant placement was nearly 4 times as likely to result in capsular contracture (p < 0.01). Rates of explantation, infection, tissue necrosis, and hematoma were comparable between groups. We also found that when subpectoral patients present with breast contracture, chemoparalysis of the muscle alone can resolve breast asymmetry, corroborating that muscle is a key contributor to breast contracture. As prepectoral breast reconstruction is gaining popularity, there have been questions regarding outcome following radiation treatment. This study suggest that prepectoral breast reconstruction is safe in an irradiated patient population, and in fact compares favorably with regard to breast contracture.

## Introduction

Breast cancer is the most common malignancy to affect women, accounting for a significant proportion of cancer-related mortality in the general female population^1^. Immediate prosthesis placement remains the leading method of reconstruction following mastectomy^2,3^. Two-stage reconstruction with tissue expander (TE) placement is most commonly used to correct mastectomy defect and restore breast form^4^. There is an increasing trend towards direct-to-implant (DTI) reconstruction in many centers, where the permanent implant is placed at the time of mastectomy in a subpectoral position, with the breast soft tissue supported along the inferolateral pole with a biomaterial surgical mesh, such as acellular dermal matrix (ADM) or synthetic material^5^.

With improvement in mastectomy skin flap viability, many surgeons have revisited implant placement in the prepectoral plane^6^. One chief factor driving prepectoral breast reconstruction is to minimize breast animation deformity that is commonly seen with subpectoral implant placement^7–9^. Placement of the tissue expander or implant within the prepectoral space reduces breast animation deformity and mitigates discomfort that may result from elevation of the pectoralis major muscle^10,11^. Early retrospective investigations have demonstrated objective cosmetic advantage following prepectoral breast reconstruction, as well as favorable patient reported outcomes when comparing prepectoral TE reconstruction to subpectoral TE placement^12–14^.

Importantly, there has been a rise in the rate of post-mastectomy radiation therapy (PMRT) in recent years^15–17^. Breast irradiation is widely reported to be an independent risk factor for post-operative complications, including surgical site infection, mastectomy skin flap necrosis, and capsular contracture^18–20^. In fact, a number of studies reported that patients who underwent PMRT following TE reconstruction were more likely to experience reconstruction failure compared to their non-irradiated cohort^21,22^. With regard to prepectoral breast reconstruction, Elswick, *et al*. reported higher rates of overall complication, including capsular contracture, although these findings were not statistically significant^23^. In contrast, Sigalove, *et al*. recently demonstrated that PMRT following two-stage prepectoral breast reconstruction was not associated with increase risk of adverse outcomes in the short-term, with average follow up of 25.1 ± 6.4 months^24^. With these conflicting reports, the impact of PMRT on capsular contracture within patients undergoing prepectoral DTI reconstruction remains unclear, as available studies are limited by sample size, heterogeneity in reconstruction methods, and lack of matched control between prepectoral and subpectoral patient groups. Additionally, most of the available data are for two-stage TE breast reconstruction and not for single-stage DTI reconstruction.

Investigation of capsular contracture as a primary safety endpoint following prepectoral breast reconstruction in an irradiated patient population serves to address a current gap in knowledge^25–27^. Radiation causes soft tissue fibrosis. In patients with subpectoral breast implant breast reconstruction, the contracture affects skin, capsule and muscle. It has been suggested that fibrosis of contractile muscle tissue could predispose patients after subpectoral reconstruction to breast contracture and implant deformation^28,29^. Evidence supporting muscle fibrosis to be a main contributor to contracture can be found in a recent investigation reporting favorable breast contracture rates following prepectoral breast reconstruction when compared to submuscular placement, in two-stage reconstruction^30^. Implant placement in the prepectoral plane avoids surgical manipulation of the muscle and the implant is not subject to deformation from muscle fibrosis and contracture following PMRT. Therefore, this study aims to compare capsular contracture rates between prepectoral and subpectoral breast reconstruction cohorts. We hypothesize that the prepectoral DTI breast reconstruction will be associated with lower incidence of capsular contracture when compared to subpectoral implant placement in an irradiated patient population.

## Methods

### Study design and population

Retrospective chart review at the tertiary academic medical center, Massachusetts General Hospital, was conducted with the approval of and in accordance to the guidelines of the Partners Healthcare Institutional Review Board. Patients who underwent immediate DTI breast reconstruction with breast irradiation, performed by the senior authors between January 2015 and May 2018, were identified. The prepectoral reconstruction group included any patient who underwent prosthesis placement following ADM or Vicryl mesh–based attachment along the anterior margin of the pectoralis major. Patient demographic, oncologic, clinical data were analyzed and pre- and post-operative clinical photographs were collected. The subpectoral group was defined as any patient who underwent implant placement within a subpectoral pocket, with inferolateral reinforcement using surgical mesh. Those patients who underwent breast reconstruction with total or partial muscle coverage were excluded from the study. Those with partial muscle coverage underwent only pectoralis major muscle dissection and tacking onto the mastectomy skin flap. Therefore, lower pole of the implant was covered solely covered by the skin flap without muscle coverage.

### Prepectoral DTI reconstruction

Following mastectomy with inframammary incision, a sizer implant was used to assess mastectomy skin flap viability and identify optimal breast implant volume. The surgical techniques employed for Vicryl or ADM prepectoral breast reconstruction by the senior author have been described previously^10,31^. In brief, a pocket of Vicryl mesh was created to enclose and support the silicon implant. If ADM was not used, the implant was fashioned to the prepectoral pocket with interrupted Vicryl sutures. If ADM was used, a piece of 18 × 8 cm was fashioned exclusively on the anterior side of the Vicryl pocket. Implant position in the prepectoral plant was controlled by suturing along the inframammary fold. The use of Vicryl mesh alone or Vicryl mesh with ADM adjunct was based on surgeon experience and technique.

### Subpectoral DTI reconstruction

Mastectomy was performed using inframammary incision. Subpectoral implant reconstruction was performed as described previously^32–34^. The pectoralis major muscle was elevated along the inferior and lateral margins. Vicryl mesh or ADM was sutured to the chest wall along the inframammary fold, and along the inferolateral border of the raised muscle. The implant was then placed within the subpectoral pocket. In this way, the superior pole of the implant was covered by pectoralis major muscle, whereas inferolateral surface of the implant was covered by surgical mesh.

### Data collection and analysis

Variables recorded for each patient were age at surgery, body mass index (BMI), obesity, history of smoking, laterality of cancer, cancer pathology, cancer grade, timing of breast irradiation, neoadjuvant chemotherapy, implant size, diabetes, mastectomy type (nipple-sparing v. skin-sparing), axillary management (axillary lymph node dissections and sentinel node biopsies), surgical adjunct (ADM v. Vicryl mesh), follow-up interval, and laterality. The primary endpoint of interest was rate of capsular contracture, defined as breast deformity resulting from either fibrous capsule formation or muscle contracture around the prosthetic device, which manifests as functional asymmetry, decreased upper extremity range of motion, and pain with activation of the pectoralis major muscle. Two blinded evaluators (LG and RE) assessed capsular contracture among a standardized set of patient photographs. Objective evaluation of the following landmarks was conducted to evaluate asymmetry^1^; axillary fold crease^2^, flattening of inferior pole projection^3^, superolateral displacement of nipple areolar complex^4^, dimpling or creasing of the soft tissue envelope at the level of the pectoralis major muscle. Superolateral displacement of the nipple was not recorded for patients with skin-sparing mastectomies. The data was then combined into a single data set, with two data points (one from each evaluator) per patient for each of the aforementioned criteria. Given the dichotomous nature of the endpoint variables, it stands to reason that blinded, independent review of a single group of patients generates two sets of *unique* data, which can be combined and subsequently analyzed. In addition, the following post-operative complications were recorded: surgical site infection, mastectomy skin flap necrosis, hematoma, revision, and explantation. Those complications that required reoperation were recorded, as surgeon management of post-operative complications varies even within a single institution. Reoperation can be objectively assessed during chart review and provides the most accurate representation of patient outcome following breast reconstruction. A Cohen’s kappa (*κ*) was calculated to determine inter-rater reliability among independent evaluators. BMI was calculated as mass/meters squared (*kg/m*^2^).

### Statistical analysis

Data were analyzed using SPSS 24 (IBM Corp., Armonk, NY). Power analysis was conducted to determine the sample size necessary to detect an effect size of 36.1%, which represents the most recently reported variance in capsular contracture rate between prepectoral and subpectoral immediate implant-based breast reconstruction in an irradiated patient population^30^. We calculated an *a priori* sample size of 62 total breast evaluations at a significance of 0.05 and a power of 80%.

Univariate analysis was conducted to compare patient characteristics between prepectoral and subpectoral groups. Normality was determined using the Shapiro-Wilk test. Normally distributed factors were compared *via* Student’s t-test and non-normally distributed variables were evaluated using the Mann-Whitney test. Fisher’s exact testing was used to compare categorical variables. Unadjusted logistic regression was used to analyze outcome data by patient. A penalized (Firth) logistic regression model was constructed to identify relationship between prepectoral breast reconstruction and post-operative complication by controlling for confounding variables (age, BMI, history of smoking, post-mastectomy radiation therapy (PMRT), mastectomy type, surgical adjunct, implant size, and post-operative follow-up). Firth logistic regression restricts maximum likelihood estimations to prevent bias that may result from small event sizes. Covariate variables that are known predictors of complication or were unmatched between patient groups were included in the regression models^35^. For each outcome endpoint, an odds ratio, 95% confidence interval, and p value were calculated. Statistical significance was defined as p < 0.05.

### Ethical approval

All procedures performed in studies involving human participants were in accordance with the ethical standards of the institutional and/or national research committee and with the 1964 Helsinki declaration and its later amendments or comparable ethical standards. Ethical approval: This article does not contain any studies with animals performed by any of the authors.

### Informed consent

Informed consent was obtained from all individual participants included in the study.

## Results

### Clinical characteristics

We identified 47 patients who underwent either bilateral (n = 34) or unilateral (n = 13) mastectomy followed by immediate reconstruction, who also had either pre- or post-mastectomy radiation treatment, resulting in a total of 81 reconstructed breasts. Although far more patients were found in the database to have underwent implant breast reconstruction and radiation treatment, these 47 patients had complete photographic documentation pre-operatively, before radiation treatment and after radiation treatment over time. Patients that lacked photographic documentation at any of these clinical junctures were excluded. Subpectoral implant-based reconstruction was performed in 27 radiated patients, whereas the remaining 20 underwent prepectoral implant placement and radiation treatment. Nipple sparing mastectomy was performed in 32 (68.1%) patients, while skin-sparing mastectomy was performed in 15 patients. The mean age of the women at time of surgery was 50.8 ± 11.3 years. Table 1 presents clinical characteristics of the total cohort and by position of implant (subpectoral v. prepectoral). Cohorts were well matched, with no statistically significant differences in patient characteristics. Mean follow-up did not vary between prepectoral and subpectoral reconstruction groups (25.3 ± 10.8 months v. 27.0 ± 11.3, respectively, p = 0.18). However, patients in the prepectoral group were more likely to be obese than those in the subpectoral group. Importantly, this variance in patient characteristics was controlled for using penalized logistic regression modeling. Oncologic characteristics, including tumor pathology, tumor grade, and TNM staging, were included in Table 1. No significant differences were observed between prepectoral and subpectoral groups.

**Table 1:** Clinical characteristics.

Variable	Total (%)	Subpectoral (%)	Prepectoral (%)	*p*
No. Patients	47	27	20	
No. Breasts	81	49	32	
Laterality				0.19
Unilateral	13 (27.7)	5 (18.5)	8 (40.0)	
Bilateral	34 (72.3)	22 (81.5)	12 (60.0)	
Mean age at surgery (yr)	50.8 ± 11.3	49.7 ± 11.0	52.3 ± 11.8	0.73
Mean BMI (kg/m^2^)	26.4 ± 5.3	24.8 ± 3.2	28.5 ± 6.6	0.08
No. Obese^†^	8 (17.0)	1 (3.7)	7 (35.0)	0.01*
History of smoking	0 (0.0)	0 (0.0)	0 (0.0)	1.00
Laterality of Cancer				0.42
Right	26 (55.3)	16 (59.3)	10 (50.0)	
Left	21 (44.7)	11 (40.7)	10 (50.0)	
Pathology				
IDC	37 (78.7)	22 (88.5)	15 (75.0)	1.00
Grade 1	3 (6.4)	1 (3.7)	2 (10.0)	0.57
Grade 2	16 (34.0)	8 (29.6)	8 (40.0)	0.54
Grade 3	18 (38.3)	13 (48.2)	5 (25.0)	0.14
ILC	2 (4.3)	0 (0.0)	2 (10.0)	0.18
Grade 1	0 (0.0)	0 (0.0)	0 (0.0)	1.00
Grade 2	2 (4.3)	0 (0.0)	2 (10.0)	0.18
Grade 3	0 (0.0)	0 (0.0)	0 (0.0)	1.00
DCIS	31 (66.0)	20 (74.1)	11 (55.0)	0.34
Grade 1	1 (2.1)	0 (0.0)	1 (5.0)	0.43
Grade 2	9 (19.2)	6 (22.2)	3 (15.0)	0.71
Grade 3	21 (44.7)	14 (51.9)	7 (35.0)	0.37
LCIS				
Grade 1	0 (0.0)	0 (0.0)	0 (0.0)	1.00
Grade 2	0 (0.0)	0 (0.0)	0 (0.0)	1.00
Grade 3	0 (0.0)	0 (0.0)	0 (0.0)	1.00
Tumor Staging				
T1	0 (0.0)	0 (0.0)	0 (0.0)	1.00
T2	23 (48.9)	14 (51.9)	9 (45.0)	0.77
T3	15 (31.9)	8 (29.6)	7 (35.0)	0.76
T4	4 (8.5)	1 (3.7)	3 (15.0)	0.30
Tis	2 (4.3)	2 (7.4)	0 (0.0)	0.50
N0	17 (36.2)	10 (37.0)	7 (35.0)	1.00
N1	23 (48.9)	12 (44.4)	11 (55.0)	0.56
N2	1 (2.1)	1 (3.7)	0 (0.0)	1.00
N3	2 (4.3)	2 (7.4)	0 (0.0)	0.50
M0	41 (87.2)	23 (85.2)	18 (90.0)	1.00
M1	1 (2.1)	0 (0.0)	1 (5.0)	0.43
ER (+)	39 (83.0)	21 (77.8)	18 (90.0)	0.27
PR (+)	33 (70.2)	17 (63.0)	16 (80.0)	0.21
HER2 (+)	13 (27.7)	9 (33.3)	4 (20.0)	0.31
Sentinel Node Biopsy	31 (66.0)	17 (63.0)	14 (70.0)	0.76
Axillary Lymph Node Dissection	16 (34.0)	10 (37.0)	6 (30.0)	0.76
Breast irradiation				0.45
Pre-mastectomy	11 (23.4)	7 (25.9)	4 (20.0)	
Post-mastectomy	36 (76.9)	20 (74.1)	16 (80.0)	
Neoadjuvant chemotherapy	56 (62.3)	13 (48.2)	14 (70.0)	0.15
Type of mastectomy				0.36
Skin-sparing mastectomy	15 (31.9)	7 (25.9)	8 (40.0)	
Nipple-sparing mastectomy	32 (68.1)	20 (74.1)	12 (60.0)	
Axillary Management				
Axillary Lymph Node Dissection				
Sentinel Lymph Node Biopsy				
Mean implant size (*mL*)	434.0 ± 159.3	407.4 ± 147.7	470.0 ± 170.9	0.17
ADM use	38 (80.9)	21 (77.8)	17 (85.0)	0.28
Follow-up time	25.3 ± 10.8	27.0 ± 11.3	22.9 ± 10.0	0.18

BMI, body mass index; TE, tissue expander; DTI, direct-to-implant; ADM, acellular dermal matrix.

*Statistically significant (*p* < 0.05).

^†^BMI >30.

### Outcomes

The most common post-operative complication was hematoma [n = 2 (4.3%)], followed infection and explantation [n = 1 (2.1%)] (Table 2). Tissue necrosis requiring surgical correction was not observed in this patient series. The rate of reconstruction revision was 14.9%, most often performed to address asymmetry from capsular contracture, to mitigate animation deformity in the subpectoral cohort, address contour irregularity, or other indications related to discomfort.

**Table 2:** Overview of postoperative complications.

	Total (%)	Subpectoral (%)	Prepectoral (%)	*p*
Overall complication	4 (8.5)	4 (14.8)	2 (10.0)	0.18
Infection	1 (2.1)	1 (3.7)	0 (0.0)	0.57
Tissue necrosis	0 (0.0)	0 (0.0)	0 (0.0)	1.00
Hematoma	2 (4.3)	2 (7.4)	0 (0.0)	0.33
Explantation	1 (2.1)	1 (3.7)	0 (0.0)	0.57
Revision	7 (14.9)	7 (25.9)	0 (0.0)	0.02*

*Statistically significant (p < 0.05).

### Position of implant (subpectoral v. prepectoral)

As indicated by univariate analysis, there were no significant differences in complication rates between prepectoral and subpectoral groups. However, the rate of capsular contracture was significantly greater in patients who underwent subpectoral implant-based reconstruction (n = 28, 53.7%), compared to those who underwent prepectoral implant-based reconstruction (n = 12, 30.0%; p = 0.02) (Table 3). Accounting for potential confounding factors in a penalized logistic regression analysis, we found that the difference in capsular contracture rates among position of implant groups was statistically significant (OR, 0.24; 95% CI, 0.08 to 0.64; p < 0.01) (Table 4). There were no other significant differences in postoperative outcomes between subpectoral and prepectoral implant-based reconstruction (Table 4).

**Table 3:** Assessment of capsular contracture rate *via* blinded review of 2D patient photographs between prepectoral and subpectoral groups.

Evaluator	Prepectoral (%)	Subpectoral (%)	p
No. Patients^†^	20	27	
Evaluator 1	7 (35.0)	13 (48.1)	0.17
Evaluator 2	5 (25.0)	15 (55.6)	0.07
Total	12 (30.0)	28 (51.8)	0.02*

Cohen’s kappa coefficient (κ) for inter-rater reliability was determined to be 0.79, which suggests moderate – strong agreement among evaluators.

^†^Number of patients per evaluation.

*Statistically significant (*p* < 0.05).

**Table 4:** Results of penalized logistic regression model for capsular contracture.

Covariate	Odds Ratio (95% CI)	*p*
Prepectoral vs. Subpectoral	0.24 (0.08–0.64)	<0.01*
Age at time of surgery	1.03 (0.99–1.08)	0.15
BMI	1.08 (0.96–1.21)	0.19
Nipple- vs. skin-sparing mastectomy	0.35 (0.11–0.99)	0.05*
Post- vs. pre-mastectomy breast irradiation	1.10 (0.32–3.96)	0.88
ADM vs. Vicryl alone	1.08 (0.30–4.09)	0.91
Implant size	1.00 (0.99–1.01)	0.10
Follow-up	0.98 (0.93–1.03)	0.44

95% CI, 95% confidence interval; OR, odds ratio; BMI, body mass index; TE, tissue expander; DTI, direct-to-implant; ADM, acellular dermal matrix.

*Statistically significant (*p* < 0.05).

### Observation of muscle contribution to capsular contracture

In patients with subpectoral breast reconstruction that suffer from breast contracture and asymmetry after radiation, there is a consistent clinical observation that the muscle is the main contributor to the contracture and pain. When these patients present for revision procedure to address breast contracture, the contracted superior pole contour is marked (dotted line) pre-operatively. The desired superior pole contour is marked 2–4 cm below (solid line), to match the superior pole contour of the contralateral non-irradiated breast. As soon as the patient undergoes induction of general anesthesia, the contracted breast mound descends from the dotted line to the desired solid line, before an incision is even made. The descent of the contracted breast mound is due to the short-acting paralytic that has relaxed the fibrotic pectoralis major muscle that was contracted over the anterior contour of the breast implant. In these patients with radiation associated capsular contracture, the mitigation of breast asymmetry by muscle relaxation provides physiologic evidence that points to the muscle as the main contributor of the contracted breast pathology. In fact, we propose that in the breast reconstruction patient population after radiation, the clinical diagnosis of *capsular* contracture is an inaccurate term to describe the breast contracture, as muscle fibrosis (not capsule) is the dominant contributing pathogenic factor.

## Discussion

Emerging studies suggest that implant placement in the prepectoral space confers aesthetic and functional benefit to patients following mastectomy, including mitigation of animation deformity and muscle spasm associated with dissection of the pectoralis major muscle^36^. In addition, recent studies demonstrate no significant difference in post-operative complication rates between prepectoral breast reconstruction and subpectoral prosthesis placement, which suggest comparable safety profiles between the two reconstruction methods^23^. However, the majority of studies evaluating post-operative complication rates between prepectoral and subpectoral breast reconstruction focus on two-staged, TE-based breast reconstruction. Given the rising trend toward DTI reconstruction, this study contributes a matched patient cohort study assessing safety clinical endpoints in the context of single-stage breast reconstruction to better guide clinical decision-making. It is reasonable to speculate that patient outcomes, such as capsular contracture, could vary with the extent of implant coverage by the pectoralis major muscle, especially within the irradiated field. Therefore, this study is unique in examining capsular contracture rates between prepectoral and subpectoral DTI reconstruction patient populations.

**Figure 1:**
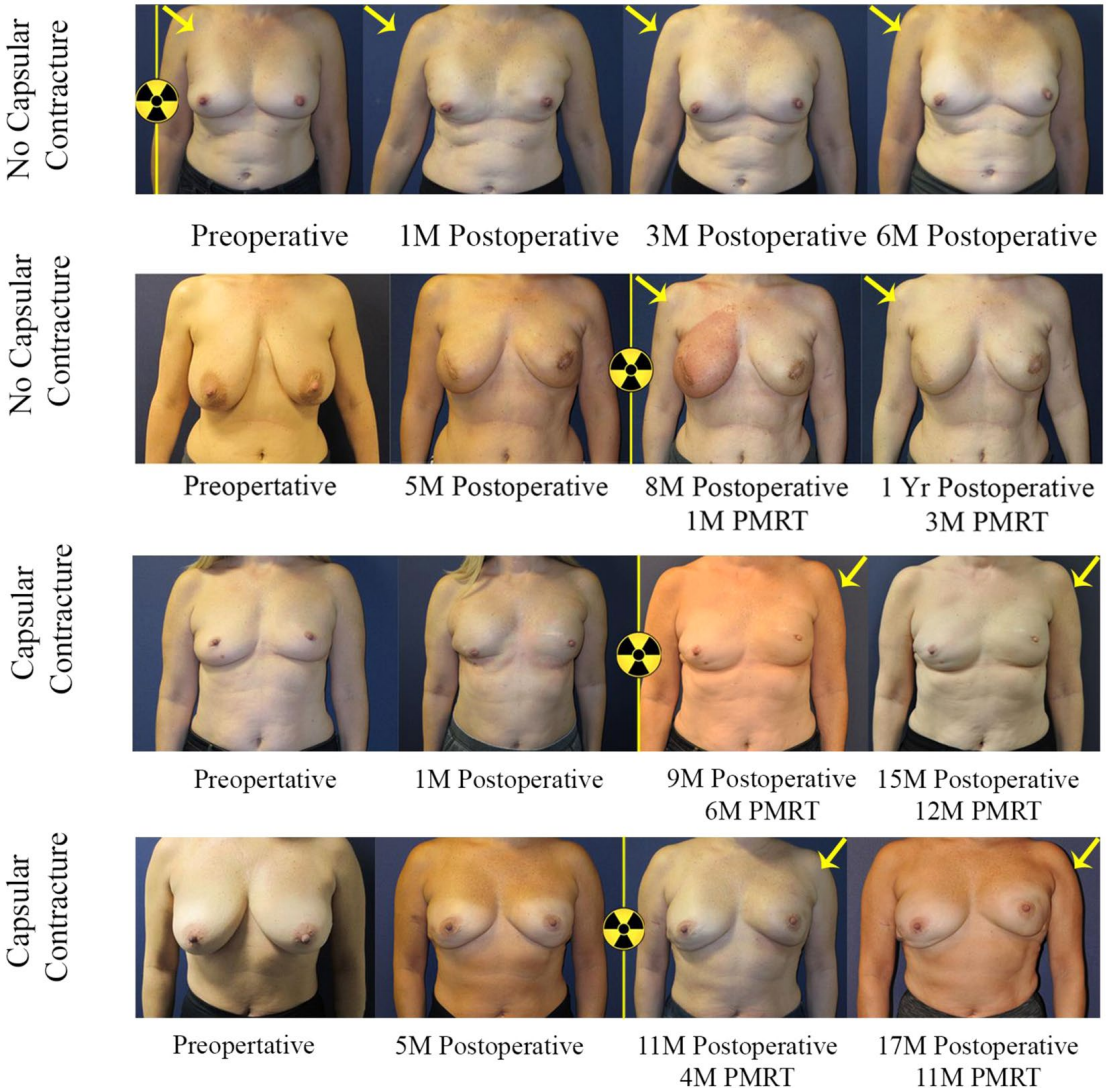
Bilateral subpectoral DTI breast reconstruction. Patient images capture signs of capsular contracture following subpectoral implant placement and breast irradiation. Patient example (row 1) received pre-operative irradiation therapy on the right breast, where breast location remained consistent with no evidence of capsular contracture. Patient example (row 2) underwent PMRT on the right breast, with no evidence of breast mound elevation or implant deformity at 3-month follow-up post breast irradiation. Patient example (row 3) with image evidence of capsular contracture demonstrated progressive elevation of the left breast. Elevation of the nipple relative to the inframammary fold can be observed concurrent with apparent breast mound rigidity, which suggests capsular contracture. Similarly, the patient image (bottom row) demonstrated elevation of the nipple and breast mound on the irradiated side compared to the contralateral, non-irradiated side. Breast asymmetry and limited prosthesis deformity were observed following subpectoral DTI breast reconstruction when compared to the control (upper rows).

**Figure 2:**
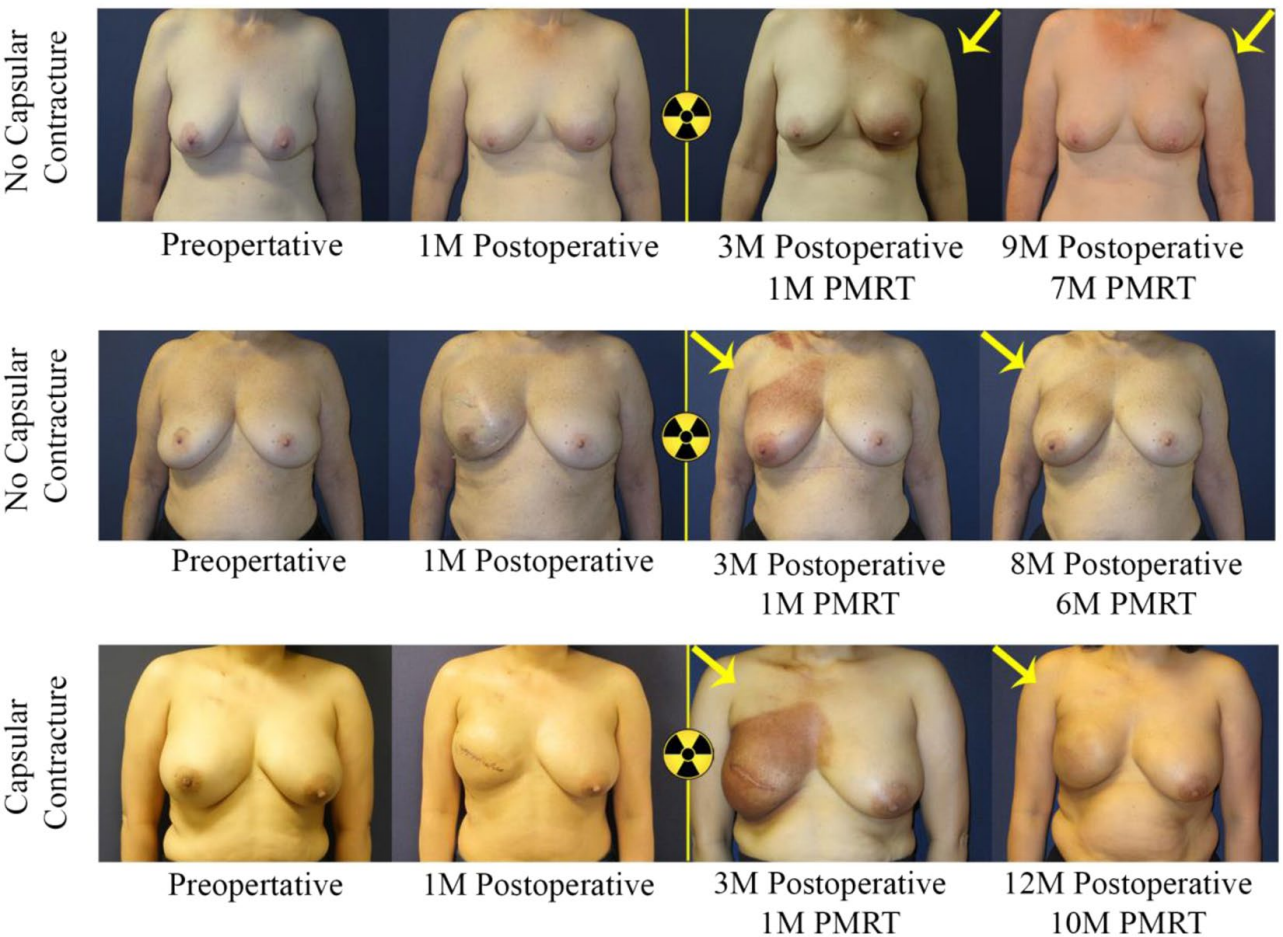
Bilateral prepectoral DTI breast reconstruction. Implant placement in the prepectoral space confers superior aesthetic result without significant prosthesis deformity or implant elevation, as evidenced by favorable nipple position relative to the inframammary fold and apparent breast symmetry. The top two patients did not exhibit signs of capsular contracture, but the bottom patient who had a tighter skin envelope on the right breast without nipple sparing, did show signs of capsular contracture. Importantly, skin dimpling or creasing is usually not observed in *contracted* prepectoral breast reconstruction, which suggests significant contribution by the pectoralis major muscle to breast mound deformity.

**Figure 3:**
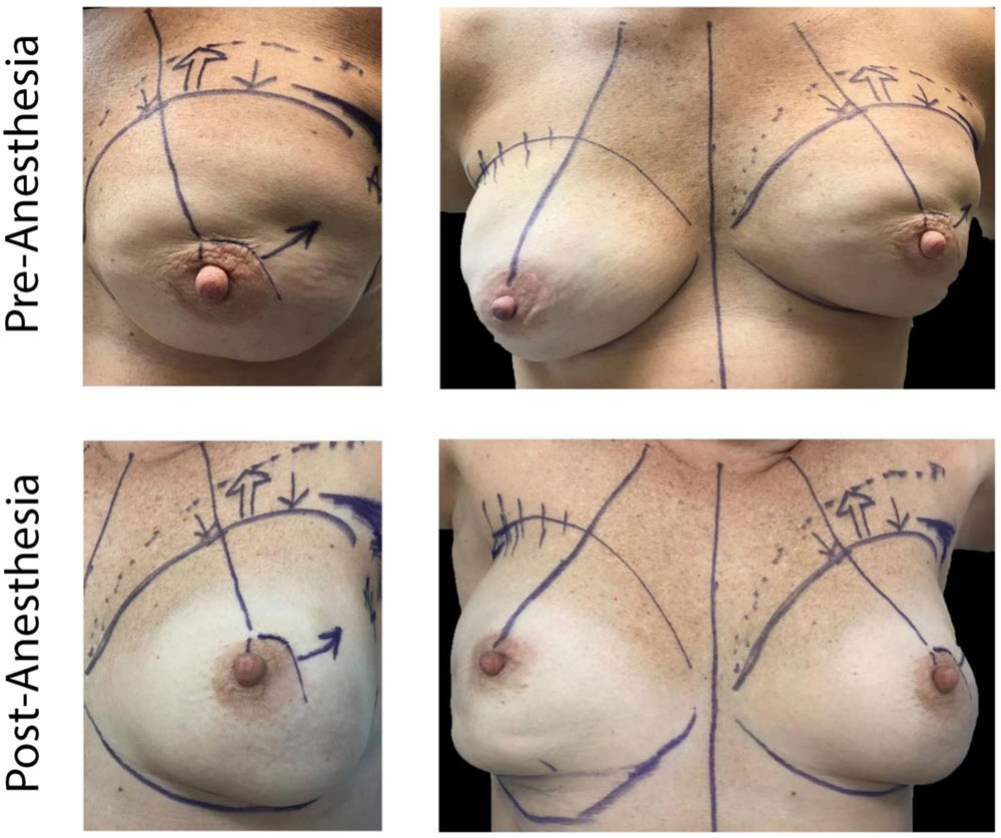
Change in implant positioning in subpectoral breast reconstruction following induction of generalized anesthesia. Relaxation of the pectoralis major muscle allows for descent of the implant from the superior margin of the mastectomy space and alignment along the inframammary fold, conferring a more natural breast shape and contour.

This study found that patients with prepectoral breast reconstruction experienced lower rates of capsular contracture by univariate and penalized logistic regression analysis. This study supports the hypothesis that implant coverage with pectoralis major muscle tissue in the case of subpectoral breast reconstruction could predispose prosthesis deformity and contracture within the irradiated field (Figs. 1 and 2). In fact, perioperative administration of generalized anesthetic in subpectoral reconstruction resulted in relaxation of the overlying pectoralis major muscle, allowing for the implant to descend and achieve a more natural contour. Figure 3 depicts this change in implant positioning before and after administration of short-acting muscle paralytic, with apparent descent of the implant from the upper pole of the mastectomy space to the desired location. In controlling for potential confounders in a penalized logistic regression model, odds of capsular contracture within patients having undergone prepectoral breast reconstruction were nearly 4 times less than that observed within the subpectoral cohort. Importantly, this result remained consistent when controlling for *timing* of breast irradiation (pre- versus post-mastectomy), which suggests that prepectoral breast reconstruction is associated with a lower rate of capsular contracture regardless of the timing of breast irradiation. What is also reassuring, is that we found that the prepectoral breast reconstruction patients did not experience increased rates of capsular contracture in DTI reconstruction, corroborating findings from other studies reporting on primarily two-stage TE cohorts (Table 5).

**Table 5:** Odds of post-operative complication by implant position (prepectoral v. subpectoral).

Complication	Odds Ratio (95% CI)	*p*
Overall complication	0.17 (0.00–1.81)	0.16
Infection	0.73 (0.01–10.73)	0.82
Capsular contracture	0.24 (0.08–0.64)	<0.01*
Explantation	0.73 (0.01–10.73)	0.82
Hematoma	0.09 (0.00–2.19)	0.17
Revision	0.03 (0.00–0.35)	0.02*

95% CI, 95% confidence interval; OR, odds ratio.

*Statistically significant (*p* < 0.05).

We performed a subgroup analysis to evaluate timing of breast irradiation on capsular contracture rates between prepectoral and subpectoral DTI reconstruction (Table 6). We observed significant difference in capsular contracture rates between prepectoral and subpectoral patient cohorts who had undergone prior breast irradiation before the index operation, with the subpectoral cohort presenting with greater rate of capsular contracture. These are patients who had undergone breast-conserving therapy with lumpectomy and whole breast irradiation, but presented with cancer recurrence, ultimately requiring mastectomy. Similarly, patients with subpectoral DTI reconstruction presented with greater rates of capsular contracture when compared to their prepectoral counterparts following PMRT, however the result did not reach significance, likely limited by small sample size. Radiation treatment following breast reconstruction could result in prosthesis deformity and breast asymmetry due to skeletal muscle fibrosis and contracture, with displacement of the underlying implant. As such, patients with implant placement in the prepectoral space are likely to avoid implant involvement following PMRT associated pectoralis major fibrosis.

**Table 6:** Comparison of capsular contracture rates between prepectoral and subpectoral groups, by timing of breast irradiation.

Evaluator	Pre-Operative Breast Irradiation			PMRT		p
	Prepectoral (%)	Subpectoral (%)	p	Prepectoral (%)	Subpectoral (%)	
No. Patients^†^	4	7		16	20	
Evaluator 1	1 (25.0)	5 (71.4)	0.24	6 (37.5)	10 (50.0)	0.52
Evaluator 2	0 (0.0)	4 (57.1)	0.19	5 (31.3)	10 (50.0)	0.32
Total	1 (12.5)	9 (64.3)	0.03*	11 (34.4)	20 (50.0)	0.23

PMRT, post-mastectomy radiation therapy.

^†^Number of patients per evaluation.

*Statistically significant (*p* < 0.05).

Few studies have directly examined capsular contracture rates following prepectoral implant placement within the context of DTI breast reconstruction. In a recent retrospective study, Sbitany *et al*. demonstrated comparable rates of post-operative complication between prepectoral and subpectoral breast reconstruction, including overall complication and capsular contracture^37^. Despite similarity in patient characteristics and radiation exposure, this study reported on patients after two-staged TE breast reconstruction, thereby limiting generalizability of their result for DTI reconstruction. More recently, Sinnott *et al*. reported greater rates of capsular contracture following subpectoral breast reconstruction when compared to prepectoral implant placement within PMRT cohorts^30^. Whereas this study provides valuable insight into the safety of prepectoral breast reconstruction in the context of radiation therapy, heterogeneity in reconstruction approaches restricts extrapolation of this study to staged TE or DTI scenarios. Their use of a Wise pattern mastectomy incision with autologous dermal flap also require a patient population with relatively large breast sizes relative to the size of reconstruction^38^. In contrast, inframammary fold incision is more commonly used for DTI breast reconstruction to accommodate all breast sizes^39–42^. In a study of safety outcomes following prepectoral breast reconstruction, it is necessary to evaluate the post-operative complications in a generalizable patient population.

**Figure 4:**
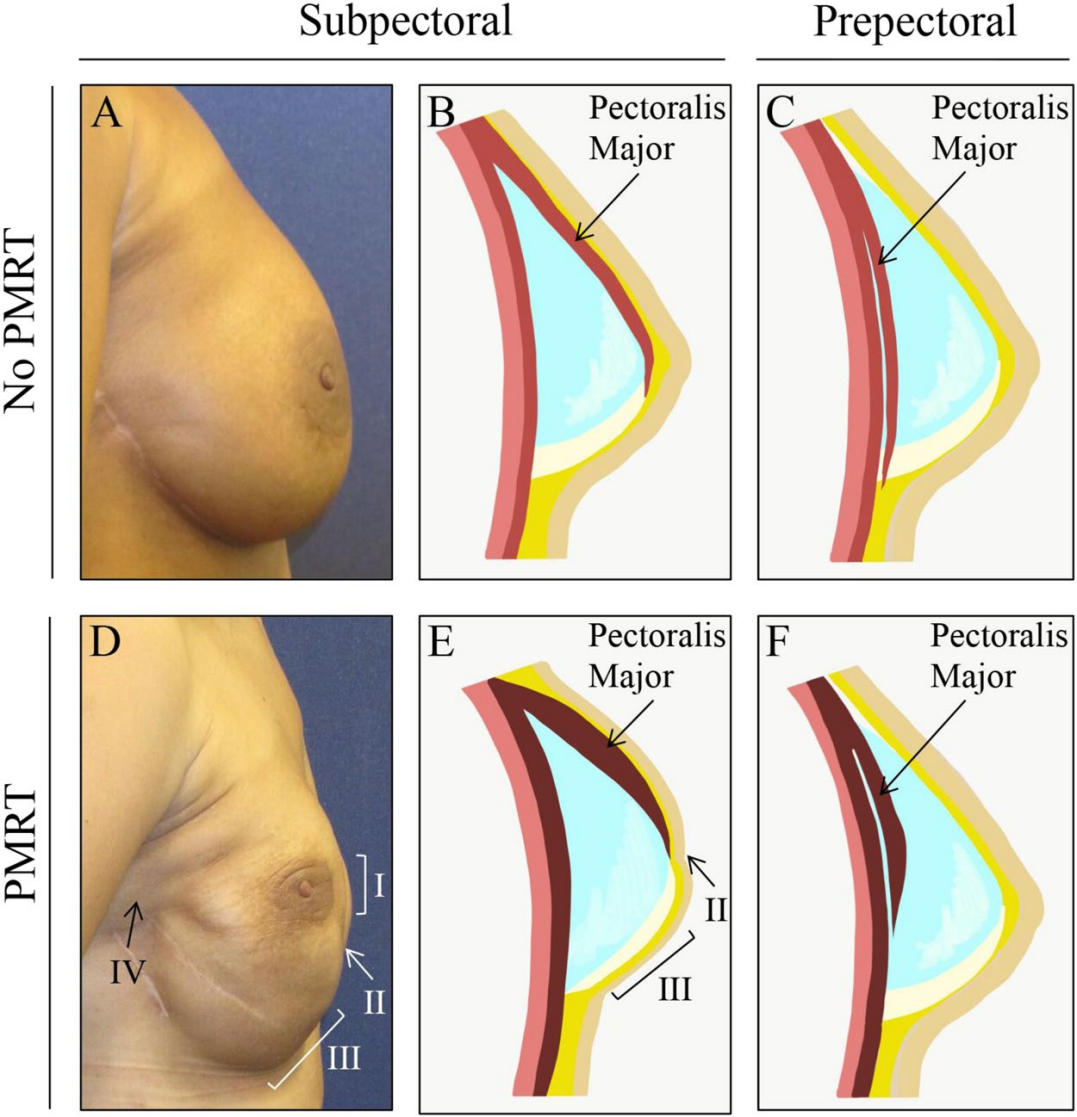
Change in breast form following post-mastectomy radiation therapy (PMRT). Panels (A–C) depict implant positioning relative to pectoralis major muscle in subpectoral and prepectoral breast reconstruction in patients who did not undergo post-mastectomy radiation therapy (PMRT). Skeletal muscle fibrosis (panels D–F) is associated with PMRT and often results in the following clinical signs after subpectoral breast reconstruction: (I) superolateral displacement of nipple areolar complex, (II) dimpling or creasing of the soft tissue envelope at the level of the pectoralis major muscle, (III) flattening of inferior pole projection, and (IV) axillary fold crease.

Analysis of capsular contracture between prepectoral vs. subpectoral implant breast reconstruction patients revealed key physical findings that we believe characterize post-mastectomy radiation soft tissue effects. The Baker-Spear classification of capsular contracture following prosthesis based breast reconstruction was published in 1995, which predates current subpectoral and prepectoral approaches that utilize surgical mesh by over a decade and is outdated^43^. We propose a classification system to describe reconstructive breast contracture that avoids the term *capsule*, as other components of the soft tissue, such as muscle, may be more significant contributors to the contracture pathology. In this analysis, we describe the following key physical findings^1^: accentuated deep axillary fold crease^2^, flattening of inferior pole projection^3^, superolateral displacement of nipple areolar complex^4^, dimpling or creasing of the soft tissue envelope at the level of the pectoralis major muscle (Fig. 4). Additionally, patients may report symptoms of animation deformity, shoulder discomfort, chest tightness, or pain. Going forward, we are using these anatomic landmarks to generate a severity classification scale for reconstructive breast contracture. For patients presenting with apparent signs of breast contracture, we are performing revision procedures to convert implant placement from sub- to pre-pectoral position, with dissection to release the pectoralis major muscle^44^.

The main limitations of this study are its retrospective nature and small sample size. Despite high inter-rater reliability, the difference in capsular contracture rate between prepectoral and subpectoral cohorts was unable to achieve significance for *each* evaluator; however, given the result of the combination group, we anticipate a trend toward significance with a larger sample. There is the potential for selection bias, as observation occurred within a single academic medical center. Additionally, inherent differences may exist in operating technique or post-operative management among surgeons, even within a single department. To mitigate variability in surgeon factors, patients were treated with a small cadre of plastic surgeons. Furthermore, variability in mastectomy skin flap thickness between prepectoral and subpectoral cohorts could introduce selection bias, as patients with sufficient subcutaneous flap thickness were selected for prepectoral reconstruction, whereas the remaining patients lacking adequate tissue thickness were selected for subpectoral reconstruction. Adequate skin thickness criteria for prepectoral reconstruction has yet to be determined in the literature and is currently based on clinical judgment to be approximately 0.5 to 1 cm in thickness, as informed by clinical experience. It would be difficult to overcome such a limitation in a retrospective study with statistical manipulation; however, prospective multi-site clinical trial with comparing prepectoral or subpectoral implant placement is currently underway to mitigate potential confounding conferred by difference in skin flap viability. Another weakness is that prepectoral breast reconstruction techniques are not standardized, so that one surgeon utilized ADM while another used Vicryl mesh. Despite these limitations, we anticipate that the results of this study showing not just safety but actually improved outcome of prepectoral vs. subpectoral implant placement are important contributions. In our practice, if we know a patient will receive post-mastectomy radiation, we will favor prepectoral breast reconstruction when possible to mitigate the occurrence of painful capsular contracture that often occurs with subpectoral implant breast reconstruction. Further, if the patient should fail implant reconstruction due to sequelae of radiation treatment, autologous breast reconstruction can be carried without the morbidity associated from prior pectoral major muscle dissection. Further, when undertaking revision surgery for patients that present with breast asymmetry, animation deformity or deformation due to radiation effect, one may consider releasing the pectoralis muscle from the implant pocket, to convert implant placement from subpectoral to prepectoral space.

The strength of this study is the comparison of capsular contracture rates between prepectoral and subpectoral DTI breast reconstruction groups in an irradiated patient population with over 18-month mean follow-up. Subpectoral breast reconstruction was associated with significantly greater rate of capsular contracture compared to prepectoral breast reconstruction by univariate analysis. This study and others debunk the notion that prepectoral breast reconstruction leads to greater capsular contracture as was the experience of the 1970s when soft tissue flaps were likely thinner and surgical mesh was not used to support the prosthetic device. Numerous studies have reported PMRT to be an independent risk factor for capsular contracture^45–47^. The result of a penalized logistic regression model suggests that PMRT is associated with a nearly 14-fold increase in rate of capsular contracture (p = 0.04). Therefore, this study confirms a known predictor of capsular contracture. Furthermore, we demonstrated no significant difference in secondary endpoints of surgical site infection, mastectomy skin flap necrosis, hematoma, revision, and explantation, supporting emerging reports that prepectoral breast reconstruction shares a comparable safety profile as subpectoral implant placement in irradiated patients.

## Conclusion

Prepectoral DTI breast reconstruction is associated with a lower rate of capsular contracture in an irradiated patient population when compared to subpectoral breast reconstruction. With continued refinement of prepectoral breast reconstruction methods, it is necessary to evaluate surgical outcomes in irradiated patient populations to improve patient outcomes in this higher risk group. We also propose that muscle fibrosis from radiation treatment is the main contributor to breast contracture and asymmetry, thus treatment of post-radiation breast contracture should address the muscle specifically.
